# Four-limb wireless IMU sensor system for automatic gait detection in canines

**DOI:** 10.1038/s41598-022-08676-1

**Published:** 2022-03-21

**Authors:** Xiqiao Zhang, Gregory J. Jenkins, Chady H. Hakim, Dongsheng Duan, Gang Yao

**Affiliations:** 1 Department of Molecular Microbiology and Immunology, School of Medicine, University of Missouri, One Hospital Dr., Columbia, MO 65212 USA; 2 Department of Biomedical, Biological & Chemical Engineering, University of Missouri, 1406 E. Rollins St. #249, Columbia, MO 65211-5200 USA; 3 Department of Veterinary Biomedical Sciences, College of Veterinary Medicine, University of Missouri, Columbia, MO 65211 USA; 4 Department of Neurology, School of Medicine, University of Missouri, Columbia, MO 65212 USA

**Keywords:** Biomarkers, Animal biotechnology, Data processing, Animal behaviour, Engineering, Biomedical engineering

## Abstract

This study aims to develop a 4-limb canine gait analysis system using wireless inertial measurement units (IMUs). 3D printed sensor holders were designed to ensure quick and consistent sensor mounting. Signal analysis algorithms were developed to automatically determine the timing of swing start and end in a stride. To evaluate the accuracy of the new system, a synchronized study was conducted in which stride parameters in four dogs were measured simultaneously using the 4-limb IMU system and a pressure-sensor based walkway gait system. The results showed that stride parameters measured in both systems were highly correlated. Bland–Altman analyses revealed a nominal mean measurement bias between the two systems in both forelimbs and hindlimbs. Overall, the disagreement between the two systems was less than 10% of the mean value in over 92% of the data points acquired from forelimbs. The same performance was observed in hindlimbs except for one parameter due to small mean values. We demonstrated that this 4-limb system could successfully visualize the overall gait types and identify rapid gait changes in dogs. This method provides an effective, low-cost tool for gait studies in veterinary applications or in translational studies using dog models of neuromuscular diseases.

## Introduction

Canine models of neuromuscular diseases have played a critical role in translating findings made in rodent models to human patients^1^. For example, Duchenne muscular dystrophy (DMD) is a lethal X-linked muscle wasting disease^2^. Results from canine DMD models have paved the way for ongoing micro-dystrophin gene therapy trials^3–6^. While canine models have a number of advantages in terms of body size, pathogenesis, immune responses, and clinical presentations, there are also challenges in using the canine model. Among these is the shortage of functional assays that can be used to accurately quantify physiological changes. Gait alterations are commonly seen in patients suffering from neuromuscular diseases^7–9^. Although the gait pattern of quadrupedal canines is different from that of bipedal humans, gait abnormalities are also a typical feature in canines affected by neuromuscular diseases^1^. Methods that can reliably evaluate gait changes in dogs are in need when canine models are used in preclinical studies.

In general, gait describes the specific form or pattern of the repeated limb movements during locomotion. Although the gait features in dogs are different from humans due to different anatomy, they still consist of periodic limb movements. One complete gait cycle of a limb is referred to as a “stride” which is classified into two separate phases: swing and stance. The swing phase describes the period when a paw is in the air, and the stance phase describes the period when the paw is on the ground. A fundamental task in gait study is to precisely determine the temporal profiles of the swing and stance, which requires the measurement of the time points when a paw steps on (swing end or stance start) or takes off (swing start or stance end) from the ground. By analyzing the temporal gait profiles from all four limbs, a specific gait type (such as walk, trot, canter, or gallop) can then be determined.

Several techniques have been used for the quantitative evaluation of canine gait. Surface electromyography was previously applied to measure gait cycles based on muscle activation patterns^10^. But the limb’s movement is intricately coordinated by multiple muscles, and electromyography’s accuracy can be compromised by off-target muscle activation. 2D and 3D video-based motion capture systems are capable of obtaining comprehensive kinematics of limb/body motion in dogs^11,12^. Unfortunately, the high cost of a complete 3D video motion system limits its wide adaptation in canine studies, and 2D video-based methods may lead to inaccurate results due to their limited view in capturing entire 3D limb movement. The pressure-sensor (aka force-plate) based “walkway” gait system is another method used to study dog gait. The “walkway” system provides an accurate 2D mapping of on-ground foot placement and vertical pressure readings but it cannot measure off-ground limb motion^13–15^.

In recent years, gait measurement using wearable wireless inertial measurement units (IMUs) has drawn a significant interest^16–30^. A typical IMU sensor can acquire multichannel motion data provided by one or more built-in microsensors, including an accelerometer, gyroscope, magnetometer, and sometimes the global positioning system (GPS)^31^. Due to their compact size, low cost, and fast 3D motion acquisition, wearable IMU sensors are ideal for free-roaming gait studies. In most previous canine gait studies^16–25^, a single IMU sensor was mounted on a dog’s main body, such as the neck, back, or sternum, which only allows assessment of general body movement. We reasoned that a sensor placed on dogs’ limbs would allow for a more precise evaluation of the gait pattern. Hence, we recently developed a canine gait detection method in which a single IMU sensor was mounted on dog’s forelimbs^30^. To fully determine a dog’s gait type, it is necessary to detect the locomotion of all four limbs.

In this study, we extended our previous forelimb IMU system into a 4-limb IMU system that can automatically characterize the complete quadrupedal gait profiles in dogs. A 3D-printed sensor holder was developed to ensure reliable and consistent mounting of the IMU sensor. New signal analysis algorithms were developed to detect the gait pattern in both forelimbs and hindlimbs. Finally, we validated the performance of the new 4-limb IMU system with a commercial pressure-sensor based walkway gait system in carefully designed synchronized tests. The results showed that our system could accurately detect the gait profiles in all four limbs of a dog.

## Methods

### Animals

All animal experiments for the study were approved by the University of Missouri Animal Care and Use Committee and were conducted according to National Institutes of Health guidelines. The proposed IMU system was tested on four 2 ~ 4 years old, mixed-breed dogs in a synchronized validation study with a pressure-sensor based canine walkway gait system (GAIT4Dog, CIR Systems, Inc., Franklin, NJ, USA). The canines used in this study were derived from cross-breeding of golden retriever, Labrador retriever, beagle, and Welsh corgi. All dogs were first trained to walk comfortably in the testing area using a leash and halter vest one month before the test. The same walk training was conducted again one week before their scheduled gait tests. All gait tests were conducted in the canine housing facility at the University of Missouri.

### Four-limb IMU sensor system

The IMU sensor (LPMS-B2, LP-Research Inc., Tokyo, Japan) has a built-in 3-axial accelerometer, gyroscope, and magnetometer in a small package of 39 × 39 × 8 mm^3^ and weights 12 g. The measurement range was set to ± 16 g (1 g = 9.81 m/s^2^) for the accelerometer and ± 2000°/sec for the gyroscope, both with 16-bit precision. The sensor sampling rate was set at 100 Hz, and acquired data was transmitted in real-time via Bluetooth to a laptop computer for processing. Only the raw data from the 3-axis accelerometer and 3-axis gyroscope were used in our signal analysis algorithm to determine the time when a paw was on and off the ground.

**Figure 1:**
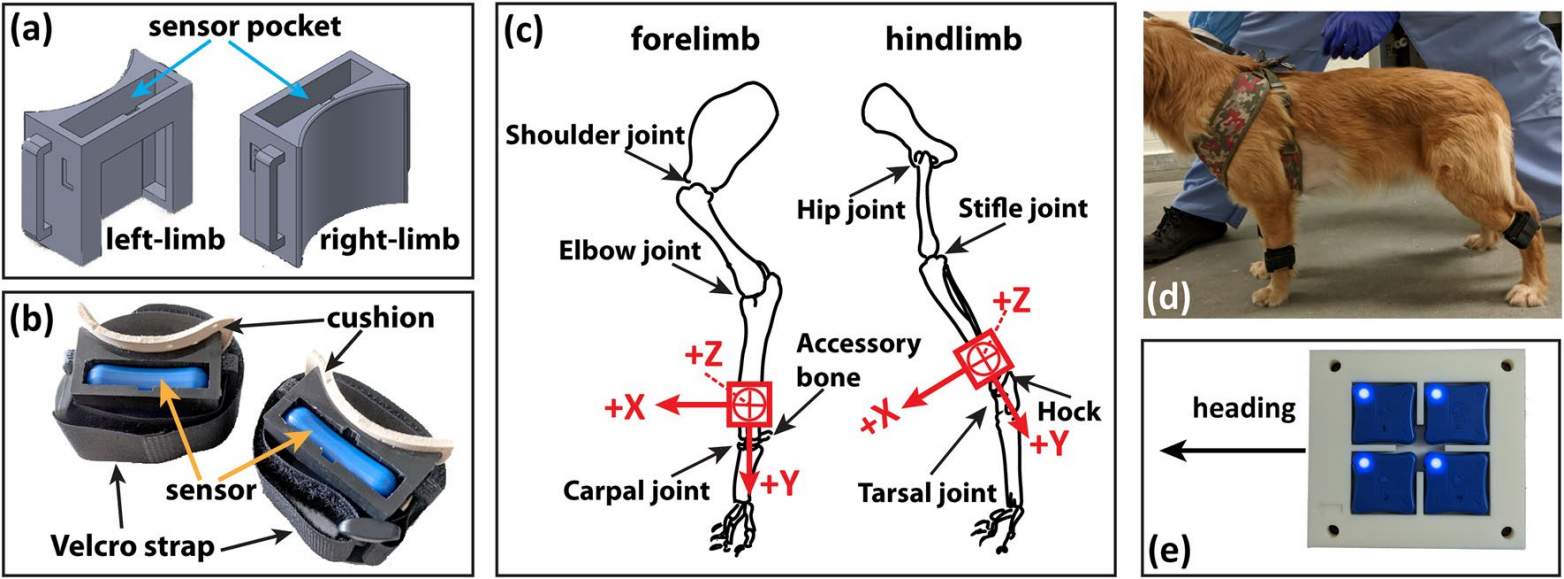
Illustration of sensor mounting and sensor/laboratory testing coordinates. (**a**) 3D-printed IMU sensor holder; (**b**) IMU sensors in holders with attached cushion and Velcro strap; (**c**) Mounting positions on the forelimb and hindlimb; (**d**) A dog with 4-limb sensors mounted; (**e**) Four IMU sensors placed in a synchronization board for heading direction synchronization.

To achieve consistent sensor mounting in tests, we designed lightweight (19 g, 55 × 24 × 42 mm^3^) sensor holders that can be 3D printed (Fig. 1a). A cushion pad (Dr. Scholl’s Air-Pillo Insole) was attached to the side of the mount in touch with the animal's limb to make the sensor mounting comfortable to the animals (Fig. 1b). The sensor holders were mounted directly above the carpal joint at the forelimb and the tarsal joint at the hindlimb using the attached Velcro straps (Fig. 1c). Both joints are easy to identify in dogs. Sensor holders were mounted on each limb by tightening the Velcro strap while the dog stands still before the gait study (Fig. 1d).

To facilitate IMU data interpretation, a coordinate system was established to describe each sensor’s position as illustrated in Fig. 1c. The x-axis of all sensors was inclined toward the heading direction (the longitudinal axis of the body). The y-axis of the sensors on the forelimbs was roughly aligned with the direction of gravity, whereas the hindlimb sensor had a slight angle in relation to the horizontal plane (ground). The z-axis is aligned with the transverse axis of the body (left–right). The sensors mounted at the left and right limbs had mirrored coordinates, which was considered during the data processing process. Our tests showed that these mounts were well accepted by the dogs and provided consistent IMU readings.

Before inserting the IMU sensor in the holders, all four IMU sensors were first placed in a tightly fitted synchronization board (Fig. 1e), turned on, and connected to the recording laptop computer via Bluetooth. After approximately aligning the synchronization board with the dog walking direction, the heading orientations of all sensors were synchronized by resetting from the control program provided by the sensor manufacturer (LP-Research Inc., Tokyo, Japan). The data streaming was started thereafter. The synchronization board was then given a quick finger tap. This quick tap left a clear signal spike in the recorded IMU signal that can be used later to check and align time bases in all four IMU sensors. After these preparation procedures, the IMU sensors were taken out from the platform and inserted into each sensor holder to start the gait tests. The recorded IMU readings were analyzed offline using a customized software to determine the time points of swing start and end events during each stride of all four limbs.

### Synchronized IMU and GAIT4Dog tests

To validate the swing/stance detection accuracy of the four limb IMU system, we conducted a synchronized test with a commercially available GAIT4Dog walkway system (CIR Systems, Inc., Franklin, NJ, USA). The GAIT4Dog system uses embedded pressure sensors inside the walkway to determine when a foot is on or off the ground. The sampling frequency of the GAIT4Dog system was set at 180 Hz in the study.

In order to align steps detected in GAIT4Dog with those identified in IMU recordings, a 120 fps GoPro camera (GoPro Hero 3 + , GoPro, Inc., San Mateo, CA, USA) was used to film the entire test. The GoPro camera was turned on before streaming the IMU sensor data. The camera recorded the finger tapping event of the synchronization board (Fig. 1e). After inserting the IMU sensors into each sensor holder on the limbs, the dog was led to stand at the starting line of the GAIT4Dog walkway. Meanwhile, the GoPro camera was positioned on a tripod behind the dog. The dog was led by the handling person to walk along the GAIT4Dog walkway at their own pace, and the leash was kept loose during the walk. The GoPro camera was positioned to capture the dog’s first hindlimb stepping event on the active area of the walkway while the GAIT4Dog kept recording the dog’s foot placement in its own system.

After the test, the recorded GoPro videos were manually analyzed to locate the image frame when the synchronization board was finger tapped. The finger tapping event was also identified in the recorded accelerometer signals of the IMU based on the signal spikes. From the GoPro video sequence, the total number of the dog’s steps was counted between sensor mounting and when one of the dog’s hindlimbs first touched on the walkway mat (referred to as “swing end” event). Using this step count, the stride corresponding to the first hindlimb “swing-end” event recorded in GAIT4Dog was identified in IMU signals.

### Validation and statistical analysis

To quantitatively evaluate the detection accuracy of swing start and end events, the following gait parameters were calculated for each identified stride:

1 $$\left\{ \begin{aligned}& {\text{Swing Duration of N}}^{{{\text{th}}}} {\text{ Stride: }}{\mathbf{SWD}}_{{\mathbf{N}}} = {\mathbf{SE}}_{{\mathbf{N}}} - \, {\mathbf{SS}}_{{\mathbf{N}}} \hfill \\& {\text{Stance Duration of N}}^{{{\text{th}}}} {\text{ Stride: }}{\mathbf{STD}}_{{\mathbf{N}}} = {\mathbf{SS}}_{{{\mathbf{N}} + {\mathbf{1}}}} - \, {\mathbf{SE}}_{{\mathbf{N}}} \hfill \\ & {\text{Stride Duration of N}}^{{{\text{th}}}} {\text{ Stride by Swing Start: }}{\mathbf{SDS}}_{{\mathbf{N}}} = {\mathbf{SS}}_{{{\mathbf{N}} + {\mathbf{1}}}} - \, {\mathbf{SS}}_{{\mathbf{N}}} \hfill \\ & {\text{Stride Duration of N}}^{{{\text{th}}}} {\text{ Stride by Swing End: }}{\mathbf{SDE}}_{{\mathbf{N}}} = {\mathbf{SE}}_{{\mathbf{N}}} - \, {\mathbf{SE}}_{{{\mathbf{N}} - 1}} \hfill \\ \end{aligned} \right.,$$

where the subscription N indicates the N^th^ stride in a single test. **SS**_**N**_ and **SE**_**N**_ represent the time points (in seconds) of the corresponding swing start and swing end, respectively. Each stride of a limb consists of a swing phase and a stance phase. The stride duration can be calculated from the swing start to the next swing start (**SDS**) or from the swing end to the next swing end (**SDE**). These four parameters in Eq. (1) were calculated using both the 4-limb IMU and Gait4Dog systems for all four limbs.

Pearson’s linear correlation was used first to evaluate the correlation between the results obtained in the two systems. In addition, the Bland–Altman analysis ^32^ was used to investigate the agreement between the measurements from IMU and Gait4Dog. Measurements from both the left and right limbs were used in the statistical analysis. Because different signal markers were used to analyze the IMU signals from the forelimb and hindlimb, these statistical evaluations were applied to evaluate the forelimb and hindlimb results separately.

All IMU signal analysis and stride phase detection algorithms used in this study were implemented in Matlab (The MathWorks Inc., Natick, MA, USA).

## Detection algorithms

### Stride detection

**Figure 2:**
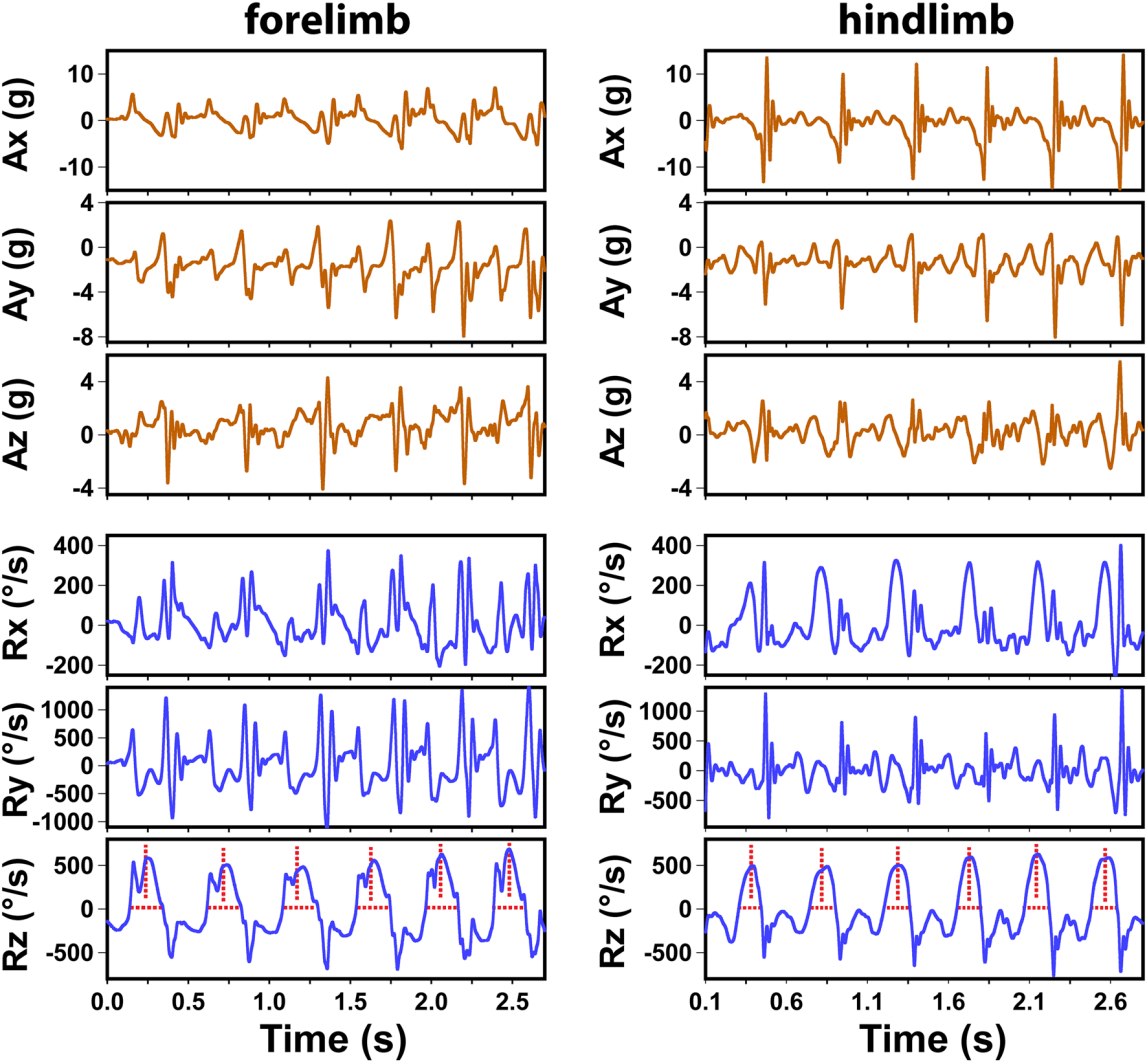
Representative IMU signals (3-axis accelerometer readings Ax, Ay, and Az and 3-axis gyroscope readings Rz, Ry, and Rz) acquired from a forelimb and a hindlimb of a dog. Vertical dashed lines mark the center of the Rz peak, and horizontal dashed lines indicate the width of the Rz peak.

The first thing to analyze IMU signals was to accurately identify all strides of each limb, which are cyclic or periodic in nature. Figure 2 shows representative raw 6-channel IMU signals from all tests recorded in this study which include 3-channel accelerometer readings (A_x_, A_y_, A_z_) and 3-channel gyroscope readings (R_x_, R_y_, R_z_). The x, y, and z directions were determined by the sensor orientation illustrated in Fig. 1. The accelerometer measures the acceleration of the sensor along the corresponding axis in units of gravity (g), whereas the gyroscope measures the sensor rotation around the corresponding axis in units of degrees per second (º/s).

Figure 2 shows clear cyclic signals in all six IMU channels for both forelimbs and hindlimbs. In comparison with the forelimb IMU signals reported in Jenkins et al.^30^, the Ax, Az, Ry, and Rz signals had similar profiles. The different appearance in Ay and Rx was largely due to the different coordinates systems used (both Ay and Rz had opposite directions). However, the hindlimb IMU signals were noticeably different from the forelimb. For example, hindlimbs had much higher Ax amplitudes, whereas their Az and Ry signals were generally smaller (Fig. 2). As discussed in Jenkins et al.^30^, the Rz peaks were also used in this study to identify each stride because the Rz signals were most consistent among all six channels for both forelimbs and hindlimbs. The strong positive Rz peaks represented the limb’s “swing” forward movement as the limb rotated around the transverse axis (the z-axis in Fig. 1).

To detect stable markers on Rz peaks, our algorithm first identified all significant peaks in Rz using the “findpeaks” function in Matlab as in the previous study^30^. From each identified Rz peak, the algorithm detected the time points when the Rz curve crossed zero on the left and right sides of the peak. The mid-point between the left and right zero-crossing was used as a timestamp marker (**T**_**N**_) of a stride, and the duration from the left and right zero-crossing was used to present the full width of the Rz peak. For the convenience of further algorithm explanation, the following two variables were defined based on the above discussion:

2 $$\left\{ \begin{aligned} &{\mathbf{T}}_{{\mathbf{N}}} :{\text{ time marker of the N}}^{{\text{th }}} {\text{stride (middle of Rz peak)}} \hfill \\ & {\mathbf{HW}}_{{\mathbf{N}}} :{\text{ half - width of the Rz peak at N}}^{{\text{th }}} {\text{stride}} \hfill \\ \end{aligned} \right..$$

Occasionally, abnormal strides may occur when a dog takes a sudden change of movement. Such strides did not follow typical gait and thus were removed in our data analysis. A stride was considered abnormal if both the width (HW_N_) and the Rz amplitude at T_N_ were outliers. A measure was determined as an outlier if its value was more than three scaled median absolute deviations (MAD) away from the median of all measurements. During a typical gait test, a typical dog would move at a relatively constant pace, then took a short pause or transition before moving again. The pausing or transitional strides were also considered abnormal. To determine such strides, all normal steps were clustered into groups by analyzing the stride duration **T**_**N+1**_−**T**_**N**_, which was the duration between two strides. A movement group ended when the next stride duration was long enough to be considered an outlier using the same MAD criterion described above. If the number of strides inside a movement group was less than three, this group was considered as transitional and thus removed from further analysis.

### Swing start and end detection

To determine the “swing start” and “swing end” time points of a stride, the six-channel raw IMU signals (Ax, Ay, Az, Rx, Ry, Rz) and their corresponding first-order time derivatives (Ax′, Ay′, Az′, Rx′, Ry′, Rz′) were analyzed to identify potential signal markers. The raw signals were interpolated to 1 kHz using spline interpolation, filtered using a 3rd order Savitzky-Golay filter (with a 0.051 s frame size) before calculating the derivatives. The Savitzky-Golay filter greatly suppressed noise in signal derivatives while preserving all significant signal features used in our feature detection algorithms. Only selected IMU signals were found useful in detecting the stride phase timings. The performance of all useful signal markers was evaluated by comparing the detection results against the references from the synchronized GAIT4Dog tests. Different signal markers were identified for forelimb and hindlimb IMU sensors due to their different IMU signal profiles (Fig. 2).

Table 1 describes the IMU signal markers with corresponding search windows. As explained in “Stride detection” section, the stride marker **T**_**N**_ was located in the middle of the corresponding Rz peak, which represented part of the swing phase of the stride. We confirmed that the swing start always occurred before **T**_**N**_**,** and swing stop occurred after **T**_**N**_. To assist in determining IMU signal markers for swing start and end, search windows were used in the algorithm to ensure correct markers were identified. For each phase time point, two markers (**M1** and **M2**) were identified to accommodate signal variations observed in strides. The second marker (**M2**) was used only when the first marker (**M1**) was not successfully detected. In regular cases, the two marker locations were very close to each other.

**Table 1:** IMU signal markers and search windows for detection of swing start and end time positions in forelimb and hindlimb strides.

	Signal markers	Search window
**Forelimb**		
Swing Start	**M1**: negative Rz’ peak with the highest prominence with positive Ax **M2**: midpoint between max(Ax) and Ax = 0	[T_N_ – HW_N_, T_N_]
Swing End	**M1**: Ax’ peak with the highest prominence excluding the first Ax’ peak **M2**: max(Rz’) outside Rz peak boundary	[T_N_ + HW_N_, T_N+1_ – 1.5 × HW_N+1_]
**Hindlimb**		
Swing Start	**M1**: Ax first crosses zero from negative to positive before the last negative Rz’ peak **M2**: Ay’ starts to decrease significantly (> 50 g/s)	[T_N-1_ + 2 × HW_N-1_, T_N_-HW_N_]
Swing End	**M1**: A_y_ reaches -g or a negative peak after peak A_y_ outside **T**_**N**_** + HW**_**N**_ **M2**: negative Rz peak after **T**_**N**_** + HW**_**N**_	[T_N_, T_N_ + 2 × HW_N_]

**Figure 3:**
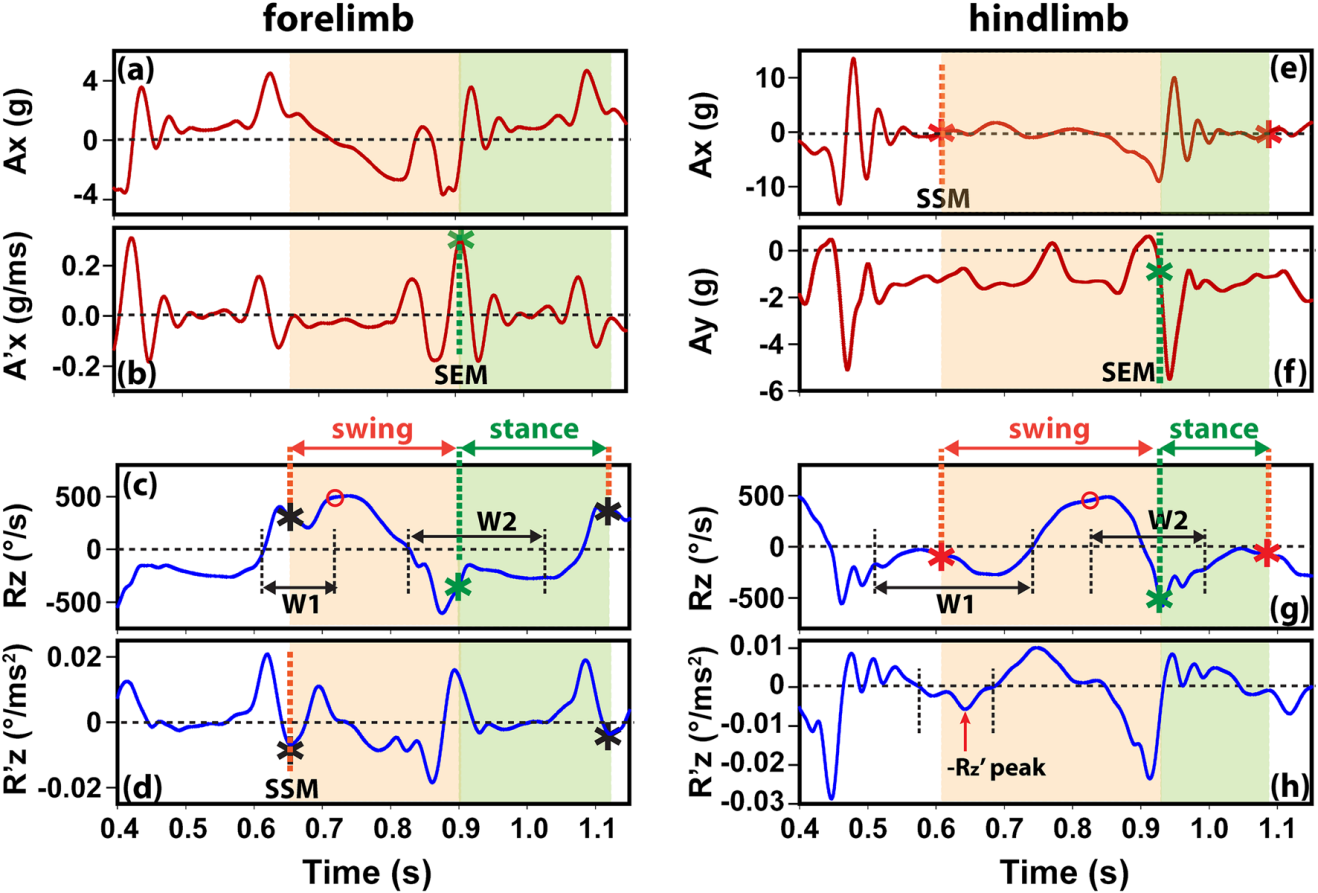
Examples of IMU signal markers (Table 1) used to detect the time points of swing start and end events in forelimb (**a**–**d**) and hindlimb (**e**–**h**). The black vertical dashed lines indicate the search window boundaries, whereas the star symbols indicate the location of the swing start marker (SSM, black/red star) and swing end marker (SEM, green star). W1 and W2 indicate the search windows for swing start and swing end, respectively.

Figure 3 shows examples of using the IMU markers (Table 1) to detect the time points of swing start and end events. In forelimbs, the swing start event always occurred between the left boundary of the Rz peak (**T**_**N**_ – **HW**_**N**_) and Rz center (**T**_**N**_), as indicated as the search window **W1** in Fig. 3c. The best signal marker for swing start (**SSM**) was the strongest negative peak in Rz′ corresponding to a positive Ax (Fig. 3d,** M1** in Table 1). A negative Rz’ with positive Ax indicated that the dog’s forelimb was accelerating in the forward direction. In rare cases when **M1** was not detected, the second marker (**M**_**2**_) was used by finding the midpoint time between maximal Ax and Ax = 0.

The forelimb swing end event occurred after the Rz peak boundary (**T**_**N**_** + HW**_**N**_) and before the start of the next Rz peak estimated using **T**_**N+1**_** – 1.5 × HW**_**N-1**_ (search window **W2** in Fig. 3c). The Ax signal approached minimal negative values at the beginning of this search window, suggesting a deceleration of the forelimb. Likely due to forces produced during deceleration, an oscillation pattern appeared in Ax and its derivative Ax′. The Ax′ peak with the highest prominence, excluding the first Ax′ peak, was a reliable marker for swing end (**SEM** in Fig. 3,** M1** in Table 1). In rare cases when a valid M1 was not identified, the algorithm used the time point of maximal Rz′ outside the Rz peak boundary as the alternative marker (**M2** in Table 1).

For hindlimbs, the swing start event of the current stride occurred after the end of the previous stride (estimated using **T**_**N-1**_** + 2 × HW**_**N-1**_) and before the left boundary of the current Rz peak **T**_**N**_**-HW**_**N**_ (search window W1 in Fig. 3g). A reliable swing start marker (**SSM**) was identified as the time when Ax crossed zero from negative to positive values (Fig. 3e,** M1** in Table 1). Such a zero-crossing event suggested the limb started to accelerate forward. We found that a valid **M1** marker had to be before the last negative Rz′ peak inside the Rz peak (Fig. 3h. If no valid **M1** was detected, the algorithm used an alternative marker (**M2**) at the time when Ay started to decrease significantly (the rate of change: Ay′ > 50 g/s).

The search window for the hindlimb swing end was defined as [**T**_**N**_, **T**_**N**_** + 2 × HW**_**N**_] (**W2** in Fig. 3g). The time point when Ay decreased to -g following a peak Ay was identified as a consistent signal marker (**M1**) for swing end (**SEM** in Fig. 3f). It is interesting to note that the Ay signal reading was -g due to gravity when the IMU sensor stayed still on the ground. This **M1** marker suggested an effective upward force produced by the touchdown impact. If no valid **M1** markers were detected, the algorithm used the location of the negative Rz peak after the right Rz boundary **T**_**N**_** + HW**_**N**_ as the alternative **M2** marker.

## Validation results and discussions

The IMU measurements were validated using 24 GAIT4Dog tests recorded in this study. For each GAIT4Dog test, four to five strides from each of the four limbs were generally recorded by the GAIT4Dog system. A dog typically took a couple of seconds to go through the active area of the walkway (4.9 m long and 0.6 wide). All strides identified in the GAIT4Dog system were matched (“synchronized) with IMU measurements as explained in the section of “Synchronized IMU and GAIT4Dog tests”. Overall, the number of synchronized swing start events detected was 237 in forelimbs and 239 in hindlimbs in the four dogs. The number of synchronized swing end events was 230 and 239 in forelimbs and hindlimbs, respectively. The four parameters listed in Eq. (1) were then calculated based on these detected events.

**Figure 4:**
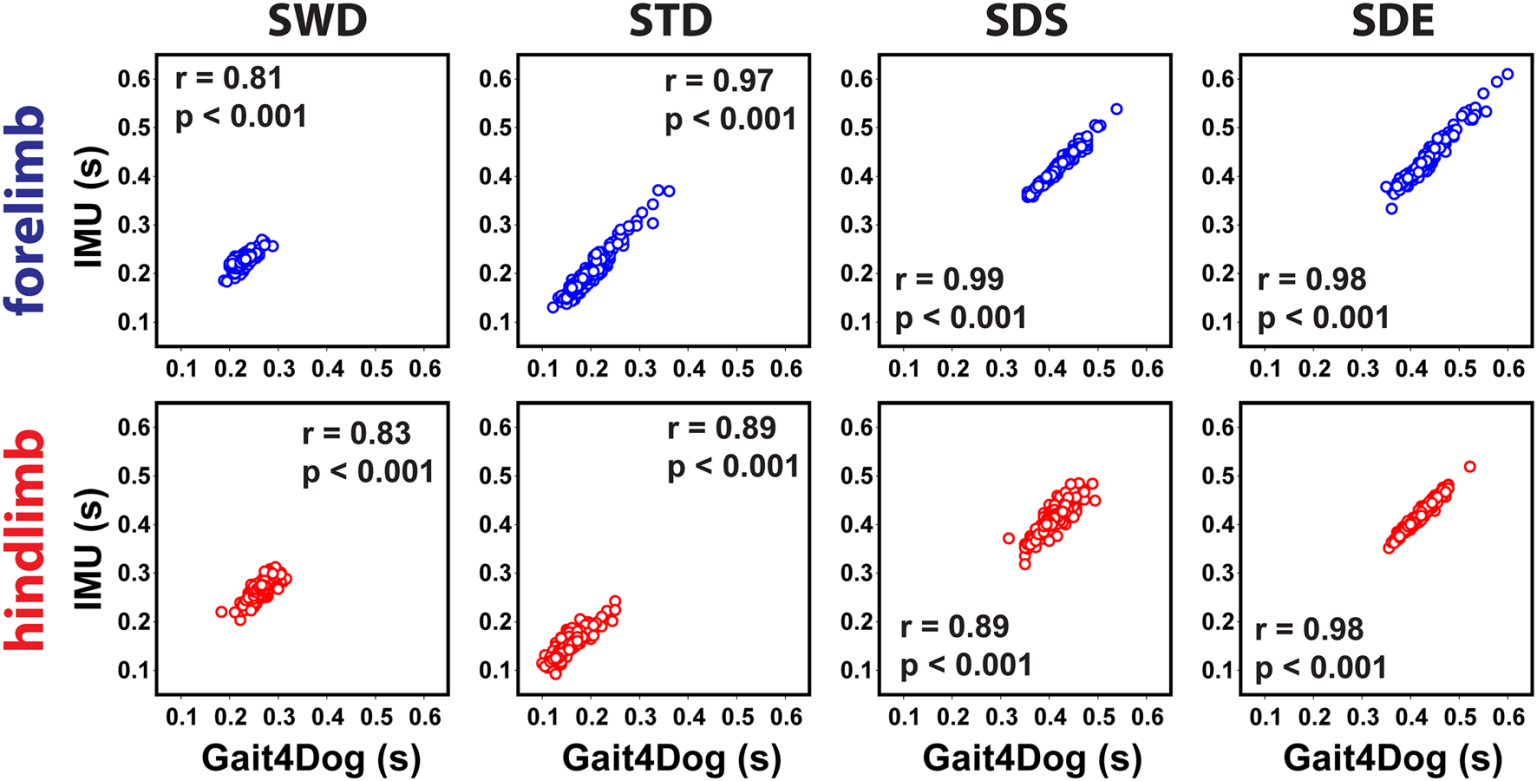
Pearson’s correlation of the four gait parameters (Eq. (1)) obtained from the IMU (vertical axes) and GAIT4Dog (horizontal axes) systems in the synchronized validation study. The correlation coefficient r and p values are given in each pairwise plot. SWD, swing duration; STD, stance duration; SDS, stride duration based on swing start; SDE, stride duration based on swing end.

Figure 4 shows the Pearson’s correlation results between IMU and Gait4Dog measurements of the four gait parameters (SWD, STD, SDS, SDE) defined in Eq. (1). All results obtained in the two systems were highly correlated (*p* < 0.001) for both forelimbs and hindlimbs. The correlation coefficient (r) ranged from 0.81 to 0.99 in forelimb and 0.83 to 0.98 in hindlimb. The correlation coefficient was equal or greater than 0.97 in STD, SDS, and SDE in forelimbs, as well as in SDE in hindlimbs. The smaller correlation coefficients in other measures were attributed to slightly greater variations between the two systems as well as the smaller data range.

**Figure 5:**
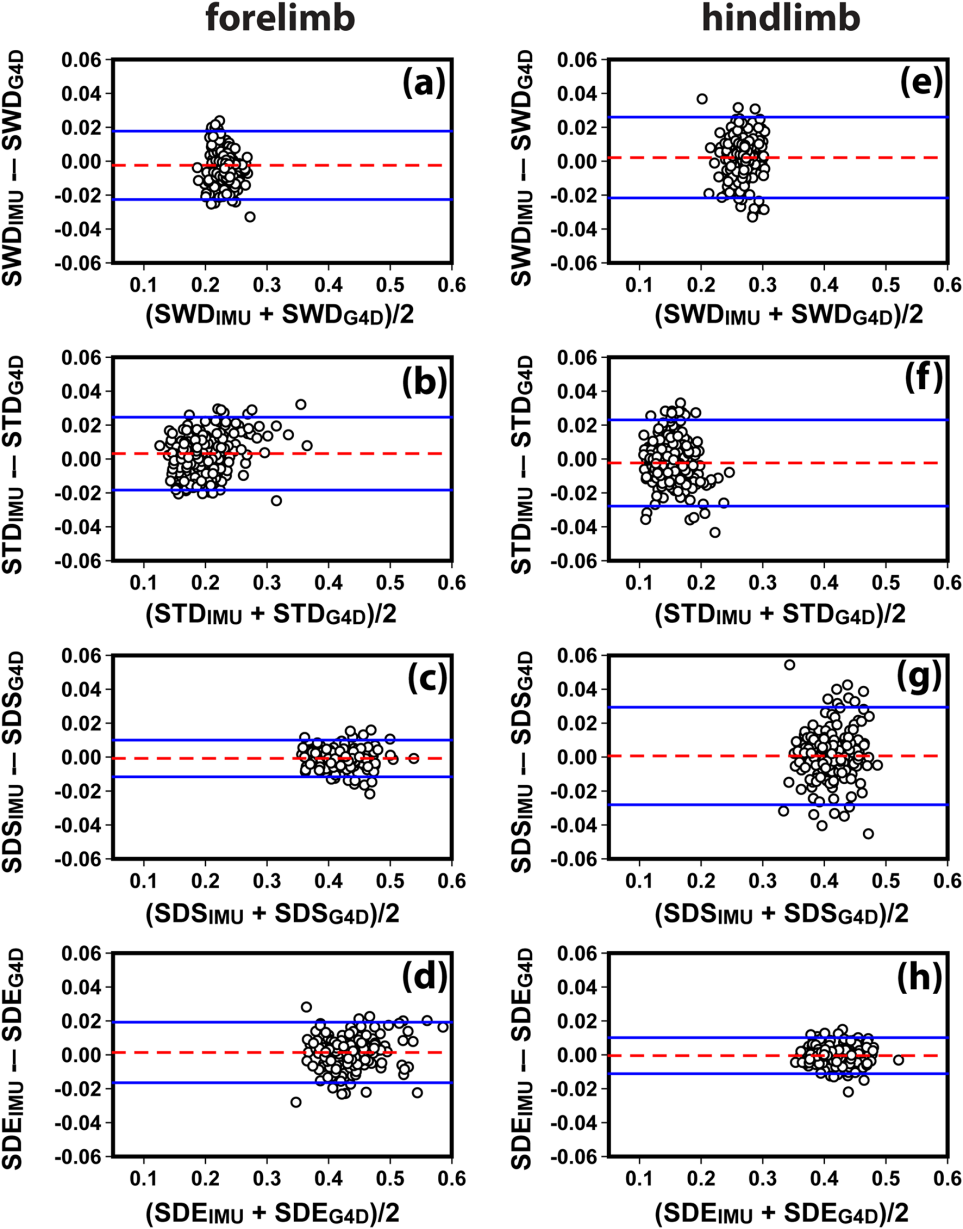
Bland and Altman plots of the four gait parameters (SWD, STD, SDS, SDE) to study the agreement between IMU (with subscript IMU) and GAIT4Dog (with subscript G4D) measurements in the synchronized validation study. The horizontal axes are the arithmetic mean of IMU and G4D readings, and the vertical axes are their difference. The measurement unit is second in all axes. The dashed lines are the mean bias between the two systems. The solid lines indicate the 95% confidence interval of the agreement. SWD, swing duration; STD, stance duration; SDS, stride duration based on swing start; SDE, stride duration based on swing end.

The Bland–Altman analysis is a reliable way to evaluate the agreement and bias between two measurement systems^32^. Figure 5 shows the Bland–Altman plots of the four gait parameters (SWD, STD, SDS, SDE) obtained from the synchronized IMU and GAIT4Dog tests. Each symbol represents a comparison of the same step parameter measured using both IMU and GAIT4Dog systems (indicated using subscript “IMU” and “G4D”, respectively). The horizontal axis is the arithmetic mean of the two measurements, and the vertical axis represents the difference between the two measurements.

In forelimbs, the mean bias (dashed line) between the IMU and Gait4Dog results was − 0.0023 s, 0.0032 s, 0.0008 s, and 0.0014 s for SWD, STD, SDS, and SDE, respectively. The corresponding 95% confidence intervals of the agreement (solid lines) were [− 0.023 s, 0.018 s], [− 0.018 s, 0.025 s], [− 0.012 s, 0.010 s], and [− 0.017 s, 0.019 s]. The disagreement between IMU and GAIT4Dog results was less than 10% of the mean value in more than 92% of the data points (97.4%, 92.2%, 100%, 100% in SWD, STD, SDS, SDE, respectively). The stride duration by swing start (SDS) had the best agreement between the two systems, which was consistent with the high correlation coefficient (r = 0.99) shown in Fig. 4.

In hindlimbs, the mean bias between the IMU and Gait4Dog results was 0.0022 s, − 0.0027 s, 0.0006 s, and − 0.0005 s for SWD, STD, SDS, and SDE, respectively. The corresponding 95% confidence intervals of the agreement were [− 0.022 s, 0.026 s], [− 0.028 s, 0.023 s], [− 0.028 s, 0.029 s], and [− 0.011 s, 0.010 s]. While the mean bias between the two systems remained low, the confidence intervals were wider than those in the forelimb comparison. Overall, the disagreement between the two systems was less than 10% of the mean value in 96.9%, 78.7%, 99.0%, and 100% of the data points in SWD, STD, SDS, and SDE, respectively. A careful examination indicated that the small percentage number of 78.7% in STD could be mainly attributed to the small mean STD values, which were the smallest among all parameters. SDE had the best agreement between the two systems with sub-millisecond mean bias and 10 ms in the confidence interval. This was consistent with the high correlation coefficient (0.98) shown in Fig. 4.

Among the four gait parameters calculated in this study, the SWD and STD values are affected by both the swing start and swing end detection (Eq. (1)). The SDS and SDE values are only affected by the swing start and swing end, respectively. Therefore, the accuracy in SDS and SDE provides a good estimation of the accuracy of swing start and swing end detection, respectively. Because the temporal resolution of the IMU system was 0.01 s (100 Hz data acquisition rate), the small limits in confidence intervals of SDS in forelimb ([− 0.012 s, 0.010 s]) and SDE in hindlimb ([− 0.011 s, 0.010 s]) suggested that the accuracy of swing start detection in forelimbs and swing end detection in hindlimbs may be close to the system limit.

Although we have conducted an extensive search for better signal markers, the phase detection accuracy of swing end in forelimbs and swing start in hindlimbs remained slightly worse than their aforementioned counterparts. It’s known that the IMU signals may be affected by variations in sensor mounting positions. The 3D printed sensor holder used in this study helped to minimize variations in mounting position. However, the IMU sensors were mounted at higher locations away from the paw to avoid affecting the dog’s movement. Because the swing start and end events were defined based on the contact between the ground and paw, the distance between the sensor and paw may ultimately affect the accuracy of detection paw-ground contact.

It should also be noted that any disagreement between the two systems may also be attributed to possible errors in the GAIT4Dog system. The walkway system is made from a large number of pressure sensors with finite sensor size and response. The paw touch events are determined based on the pressure readings using a proprietary algorithm. It’s expected that pressure readings may be affected by specific paw position, contact size, and contact speed, which can lead to fluctuations in the detection of swing start and end events. We had encountered several occasions when a dog’s steps were detected by the IMU system but not recognized by GAIT4Dog on the walkway.

Nevertheless, the overall good agreement between the IMU and GAIT4Dog systems suggests that the wireless 4-limb IMU system may be able to characterize the gait types of the dogs used in this study. In general, a canine gait can be classified as “walk”, “amble”, “trot”, “canter” etc., based on the on-ground paw patterns. For example, in “walk”, three paws are on-ground, and the other is off-ground. The off-ground paw (in swing phase) may alternate from the forelimb to the hindlimb, and from the left limb to the right limb. In “amble” gait, the on-ground paws may alternate between two limbs on the same side and two limbs in diagonal. “Trot” is a slightly faster gait and is characterized by two diagonal limbs swinging in unison while the other two are on the ground.

**Figure 6:**
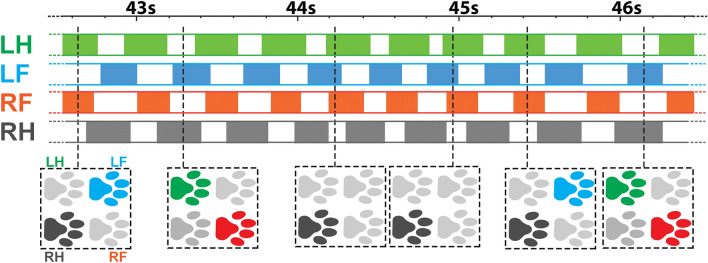
An example of time-resolved gait type detection using the 4-limb IMU system. The solid-color boxes in the top panel indicate the swing phase of a limb. The white space between two solid-color boxes indicates the stance phase (on-ground paw). In the lower panel, the pawprint in light gray represents off-ground paws, and the pawprint in solid colors represents on-ground paws. LH: left hindlimb, LF: left forelimb, RF: right forelimb, RH: right hindlimb.

In reality, a dog’s gait sequence is often complicated with mixed gait types. The top panel of Fig. 6 shows a continuous temporal “box diagram” of a stride sequence constructed using the swing start and end data measured from a dog’s four limbs. The solid boxes represent swing phases of a limb, and the stance phases are represented by the white spaces between two solid boxes. The pawprint diagrams at the lower panel visualize the on-ground paw patterns at a specific time where off-ground paws are represented in light-gray, and on-ground paws are shown in solid colors.

A quick overview of the temporal box diagram suggests that many strides can be characterized as “trot” due to the alternating diagonal on-ground paw patterns. This is most obvious at the beginning and toward the end, as evidenced in the paw diagrams around 43 s and 46 s. However, deviations can be observed in the middle part of the stride sequence. The dog appeared to speed up from ~ 44 s to ~ 45 s, which can be corroborated by smaller stance durations (white spaces between boxes in solid colors) in all four limbs. The two paw diagrams close to 44 s and 45 s show only the right hind paw on-ground, and all other three paws are off-ground. Such a paw pattern only exists in the traditional “canter” or “gallop” gait. It is interesting to note that the diagonal “trot” gait can still be observed in the middle part of the stride sequence. Therefore, we can conclude that this dog’s gait type changed rapidly during the speed-up period from the beginning toward 44–45 s and during the slow-down period thereafter.

In conclusion, we developed a 4-limb wireless IMU system for canine gait analysis. The IMU sensors can be easily mounted to a dog’s limbs using a 3D printed sensor holder, which showed no adverse impact on dog’s movement. Through intensive searching and validation, effective IMU signal markers were identified for detecting the swing start and end events in both forelimbs and hindlimbs. The system was tested on four dogs in a synchronized study using a commercially available GAIT4Dog system as a comparison reference. Owing to the novel sensor mounting system and robust signal processing algorithms, we achieved a great agreement between the measurements obtained in the IMU system and the GAIT4Dog walkway. We demonstrated that this 4-limb system was able to reveal complicated gait patterns in detail.

To the best of our knowledge, this is the only IMU based wireless gait system that can autonomically detect stride parameters in all four limbs of a dog with precision comparable with a GAIT4Dog system. With its low cost, lightweight, and wireless capability, this 4-limb IMU based gait analysis system is well positioned for applications on free roaming animals. It enables new research opportunities in studying the sophisticated coordination among all four limbs in dogs. Such a system may also be adapted for gait applications in other quadrupedal animals. Once fully developed, such a tool would be valuable for animal gait analysis at veterinary clinics as well as in translational research involving canine models (and potentially other quadrupedal animal models) of human diseases.

## Data Availability

Data are available from the corresponding authors upon reasonable request.
